# Interinstitutional beam model portability study in a mixed vendor environment

**DOI:** 10.1002/acm2.13445

**Published:** 2021-10-13

**Authors:** Sean P. Frigo, Jared Ohrt, Yelin Suh, Peter Balter

**Affiliations:** ^1^ Department of Human Oncology University of Wisconsin‐Madison School of Medicine and Public Health Madison Wisconsin USA; ^2^ Department of Radiation Physics University of Texas MD Anderson Cancer Center Houston Texas USA

**Keywords:** beam model, commissioning, parameter value optimization, portability, RayStation, TrueBeam, validation

## Abstract

A 6 MV flattened beam model for a Varian TrueBeamSTx c‐arm treatment delivery system in RayStation, developed and validated at one institution, was implemented and validated at another institution. The only parameter value adjustments were to accommodate machine output at the second institution. Validation followed MPPG 5.a. recommendations, with particular attention paid to IMRT and VMAT deliveries. With this minimal adjustment, the model passed validation across a broad spectrum of treatment plans, measurement devices, and staff who created the test plans and executed the measurements. This work demonstrates the possibility of using a single template model in the same treatment planning system with matched machines in a mixed vendor environment.

## 1 INTRODUCTION

A mixed vendor environment (MVE) for radiation therapy provides the potential to combine best‐in‐class tools while meeting each institution's technology preferences and needs. This environment is comprised of an image acquisition system (IAS, simulator), treatment planning system (TPS), treatment management system (TMS), and treatment delivery system (TDS, Linac), all working together. To cover a broad range of delivery platform technologies, TPS vendors must support generalized machines, for example, a c‐arm TDS. Without direct access to a specific TDS technology and specifications, they must code to a generalized interface, and so an MVE comes at a cost in terms of potential integration challenges and added validation burdens. The TPS and TDS are developed and validated independently from one another by the different vendors. An example of this situation is with the RayStation TPS and a Varian TrueBeamSTx TDS.

There is a broad spectrum of reported RayStation machine model parameter values in use.^1^ This lack of consensus reflects a number of things. First, the software, compared to other broadly used TPSs, is relatively new. Second, the size of the nascent user community has a correspondingly smaller collective knowledge and experience. Third, there has been variation in the interpretation of the multi‐leaf collimator (MLC) model parameter values as well as variation in input measured data. Though use has increased, and a number of RayStation machine models have been published,^2^, ^3^, ^4^ the results vary, consistent with a study by Imaging Radiation Oncology Core (IROC).^1^ Consequently, there still is not a readily available optimized RayStation model template for matched machines, although Hansen and Frigo demonstrated that this should be feasible.^5^

Building a fully validated machine model with a new TPS from scratch is a formidable task, involving data collection, parameter value optimization, and dosimetric validation. The Medical Physics Practice Guideline for Commissioning and QA of Treatment Planning Dose Calculations (MPPG 5.a.) cites reasonable time estimates of 2–4 weeks to commission a single energy photon beam, considering 12–16 h per day of 1.5–2.0 full‐time‐equivalent qualified medical physicist effort.^6^ First, the physicist must learn about the approximations and assumptions in the software. This is a challenge, as many clinical physicists do not have the time and background to achieve the intimate understanding needed in order to optimize model parameter values, especially for dynamic MLC beams. Second, the amount of work in creating a broad spectrum of test beams that cover a clinic's treatment approaches, executing measurements with those plans, and performing an analysis of the results, is significant. Much needs to be done outside of the TPS, using third‐party or home‐built tools. Compounding this effort are variations in measurement equipment as well as in their use. Errors in model construction are well documented.^7^

Jacqmin et al. presented an example implementation of the MPPG 5.a. guidance for two different TPSs (Pinnacle and Eclipse).^8^ Each TPS was tested in the context of a single institution, and focused on MPPG 5.a. implementation aspects, including analysis tools. Model performance across matched TDS and detailed intensity modulated radiation therapy (IMRT)/volume modulated arc therapy (VMAT) results across a broad spectrum of treatment plans were out of that work's primary aim. To our knowledge, there are no comprehensive MPPG 5.a. photon validation studies that span multiple institutions for the same TPS machine model for a mixed‐vendor environment of the respective systems (IAS, TPS, TMS, and TDS).

Single vendor environments (SVEs) help address integration and validation challenges by providing bundled solutions. To bring some of the advantages of an SVE into an MVE, we present the results of a broad MPPG 5.a. validation of a single machine model, demonstrating MVE model portability for the first time. This was performed at two completely independent institutions, using different types of measurement equipment, multiple personnel, and multiple TDS instances that all meet a common vendor‐defined machine performance specification.

As designs have evolved, and with advances in manufacturing processes and technology, modern TDSs now exhibit a consistent standard of performance.^9^, ^10^, ^11^ This makes it feasible to establish conformance to a single beam performance specification for each beam energy/modality. This has enabled the results of this work, which demonstrate the potential to use an unmodified RayStation machine model with any appropriately matched machine, without any need for further model parameter value optimization. Under these circumstances, the physicist can proceed directly to end‐to‐end validation testing. A type‐tested MVE template model only needing validation, heretofore only available in SVEs, is a benefit to the community.

## 2 METHODS

### 2.1 Treatment delivery systems

This study focuses on a single TDS class, the Varian TrueBeam with a high‐definition multi‐leaf collimator (TBSTx) (Varian Medical Systems, Palo Alto, CA, USA). Institution A has one TBSTx (Linac A1), and Institution B has two (Linac B1 and Linac B2). At each institution, the Linacs were demonstrated to pass standard vendor acceptance testing procedures and met the same performance specifications, including meeting the vendor's Enhanced Beam Conformance specifications.^12^, ^13^ In addition, standard beam commissioning data, including output factors, percent depth‐dose, and profiles, were compared to data in the literature from other institutions.^11^

### 2.2 Treatment planning systems

Both institutions used the RayStation (RaySearch Laboratories, Stockholm, Sweden) TPS for test plan creation and dose calculation using a collapsed‐cone convolution dose calculation algorithm. Institution A used version 7.0.0.19 (RayStation 7) with the CCDose 3.5 dose engine, and Institution B used version 8.0.1.10 (RayStation 8A SP1) with the CCDose 4.1 dose engine. Between these two versions, there were two updates to the CC Dose algorithm.^14^ The first update had no effect on the TBSTx Linac class. However, it did require the beam model to be recommissioned in the software when upgrading. The second update affected the dynamic MLC (DMLC) fluence calculation and fixed an issue with rotated collimators and asymmetric primary sources.^14^ The second update is characterized as minor, and does not require a beam model to be recommissioned when upgrading.

For Institution A, a TBSTx class machine was defined in the TPS, using vendor specifications for all mechanical properties. Dose engine (model) parameter values were determined by a three‐step process. First, non‐MLC parameter values were optimized using jaw‐defined beam measurement data using the TPS modeling tools within the RayPhysics module of the TPS. Second, the MLC parameter values were initialized using values from ray‐tracing performed outside of the TPS. Then, MLC parameter values were further optimized to obtain best agreement with ion chamber measurement of VMAT deliveries. The fluence parameter values of the model are summarized in Table 1.

**TABLE 1:** Institution A fluence parameter value summary

Parameter	Value
Primary Source X‐width (cm)	0.060
Primary Source Y‐width (cm)	0.045
MLC X Offset (cm)	0.013
MLC X Gain	0.000
MLC X Curvature (cm^–1^)	0.000
MLC Leaf Tip Width (cm)	0.250
MLC Transmission	0.0115
MLC Tongue Groove Width (cm)	0.040

The Institution A model was then validated using AAPM Medical Physics Practice Guideline 5.a.,^6^ for static, step‐and‐shoot (SAS) IMRT, and volume modulated arc therapy (VMAT) delivery techniques following the formalism of Jacqmin et al.^8^ The resulting TBSTx machine with Institution A parameter values was then considered as the candidate base model for portability testing.

A copy of the Institution A model was then provided to Institution B. Institution B verified that all nondosimetric parameters were valid for its TBSTx machines. To ensure that this model would be representative of the Institution B clinical standards and measured data, all of Institution A's measured beam data were removed and replaced with Institution B's. This entailed all percent depth dose, profile, and output factor entries.

Institution A's 6 MV absolute calibration coefficient value of 0.664 cGy/MU was changed to match Institution B's in‐house absolute dose specification of 0.667 cGy/MU. The Institution A Dose Normalization factor of 3.8338 was changed to 3.8541 at Institution B to accommodate the slightly different calibration coefficient value. Institution B's measured output factors were slightly different than institution A's and it was decided to recompute the output factor corrections (OFCs). These output‐related changes were the only site‐specific updates to the RayStation machine. The different values and their ratios are summarized in Tables 2 and 3.

**TABLE 2:** Output factor values

Field	Output factor		
size	Institution	Institution	A/B
(cm^2^)	A	B	ratio
1x1	0.7056	–	–
2x2	0.7902	–	–
3x3	0.8336	0.8300	1.0043
4x4	–	0.8640	–
5x5	0.8962	0.8950	1.0013
6x6	–	0.9210	–
8x8	0.9662	0.9660	1.0002
10x10	1.0000	1.0000	–
12x12	–	1.0280	–
15x15	1.0588	1.0610	0.9979
20x20	1.0995	1.1010	0.9986
25x25	–	1.1300	–
30x30	1.1511	1.1540	0.9975
35x35	–	1.1740	–
40x40	1.1729	1.1890	0.9865

**TABLE 3:** Output factor correction values

Field	Output factor correction		
size	Institution	Institution	A/B
(cm^2^)	A	B	ratio
1x1	0.9879	–	–
2x2	0.9894	–	–
3x3	1.0010	0.9974	1.0036
4x4	–	1.0006	–
5x5	1.0037	1.0010	1.0027
6x6	–	1.0027	–
8x8	1.0042	1.0016	1.0026
10x10	1.0000	1.0000	1.0000
12x12	–	0.9973	–
15x15	0.9979	1.0010	0.9969
20x20	1.0050	1.0042	1.0007
25x25	–	1.0097	–
30x30	1.0107	1.0136	–
35x35	–	1.0187	–
40x40	1.0078	1.0252	0.9830

The beam profile and depth curves were recomputed in the TPS physics module and compared with Institution B's measured data as an initial validation step. Institution B independently validated the model using MPPG 5.a. The testing included all model performance specifications for static, SAS, and VMAT delivery techniques.

### 2.3 Treatment management systems

Each institution employed different TMS. Institution A utilized ARIA 13.6 (Varian Medical Systems, Palo Alto, CA, USA), while Institution B used Mosaiq 2.65 (Elekta, Stockholm, Sweden). All TPS plan data were exported from the institution's RayStation TPS instance to the TMS and then to their respective TDS for delivery. The integrity of the data chain was validated by standard end‐to‐end testing procedures.

### 2.4 Dose validation

To meet the testing recommendations in MPPG 5.a, both institutions used commercially available measurement systems for IMRT/VMAT deliveries. In total, four different devices were employed, each of a different design, two at Institution A and two at Institution B. All were calibrated per vendor procedures to produce absolute dose readings, using completely different ADCL‐calibrated ion chambers present at each institution.

Institution A employed a basic VMAT test plan suite comprised of four geometrically based TG‐119 and three anatomically based patient care plans.^15^ A second broader suite of 24 plans was created based on earlier clinically delivered plans using institutional protocols and optimization techniques, designed to span the potential spectrum of potential treatment scenarios. Beam sets created from plans derived from anatomically based geometries were limited to targets 3 cm (15 cm^3^ volume) diameter or larger. These specific tests did not consider smaller targets, for example, for stereotactic radiosurgery (SRS) delivery techniques.

A Tomo “Cheese” phantom (Accuray, Sunnyvale, CA, USA) with an array of six A1SL (Standard Imaging, Middleton, WI, USA) ion chambers was used at Institution A to measure the basic VMAT plans. All detectors were located in the low‐gradient high‐dose regions within target volumes. Estimated uncertainty in the A1SL dose values was 1%.^16^ All measurements were corrected for machine output. A local dose percent difference (PD) was calculated between the derived A1SL ion chamber dose measurement (M) and corresponding ROI average dose (C) in the RayStation TPS. The percent difference was defined as 100 * (M – C) / M.

Institution A also utilized a Delta4‐Plus diode array (Scandidos, Uppsala, Sweden) for the broader suite of test plans.^17^ Both a 3D gamma and median dose difference (MDD) analyses were performed for each measurement. The MDD is defined as:

$$MDD=med\left(\left\{\left(M_{i}-C_{i}\right)/M_{i}\right\}\right),\;i=1,\ldots ,N$$

for the distribution of *N* measured (*M_i_*) and calculated (*C_i_*) dose pairs, respectively, and expressed as a percentage. Gamma analyses utilized both current clinical levels of 3% global percent difference (GPD), 3 mm distance to agreement (DTA), and 20% dose threshold (DT), as well as tighter levels of 2% local percent difference (LPD), 2 mm DTA, and 20% DT. The Delta4 absolute dose measurement error is estimated to be 1%. All Delta4 measurements were corrected for machine output.

Institution B measured a cohort of 60 clinically based plans representing the range of deliveries on their two TBSTx machines. These plans include SAS IMRT and VMAT, cover multiple treatment sites, and are generally organized into three categories: stereotactic spinal radiosurgery (SSRS), stereotactic body radiotherapy (SBRT), and nonstereotactic.

An Octavius 4D ion chamber array (PTW, Freiburg, Germany)^18^ was used for SSRS deliveries. A gamma analysis was performed using clinical criteria (4% LPD, 2.5 mm DTA, and 30% DT), as well as with more stringent ones (2% LPD, 2 mm DTA, and 30% DT). In addition to gamma analysis, the MDD as defined above was also recorded. All measurements were corrected for machine output.

An ArcCHECK (Sun Nuclear, Melbourne, FL, USA) diode array was used for all other cases (SBRT and nonstereotactic).^19^, ^20^ Clinical analysis criteria (3% GPD, 3 mm DTA, and 10% DT) were used when comparing measured dose at the detectors versus that calculated by the TPS. The ArcCHECK software does not report an MDD, and it was not possible to reanalyze the dose distribution with stricter gamma criteria (2% LPD, 2 mm DTA, and 10% DT). All measurements were corrected for machine output.

Both institutions currently employ more relaxed gamma criteria than recommended in the TG‐218 report (3% GPD, 2 mm DTA, and 10% DT),^21^ hence, the inclusion of analyses using stricter criteria when possible (2% LPD, 2 mm DTA, and 20% DT).

### 2.5 Independent audit

Both institutions participated in an independent audit using the anthropomorphic SBRT Lung phantom provided by the IROC.^22^ The phantom contains dosimeters that measure absolute dose at a few points, film that measures relative planar dose, as well as a localization assessment. The phantom was scanned, planned, and treated by each institution being audited and returned to IROC for analysis and comparison with the 3D dose distribution from the TPS using point dose local percent difference (dosimeters) or a 2D gamma analysis (film).

## 3 RESULTS

The results are presented by institution, with the validation performed at Institution A first. Following thereafter are the changed model parameter values at Institution B and their subsequent validation results.

### 3.1 Institution A

The results for Institution A are presented in the following subsections. This includes MPPG 5.a. static beams with a 3D tank, and VMAT plans with a Tomo “Cheese” phantom as well as a Delta4 device.

#### 3.1.1 MPPG 5.a. summary

All MPPG 5.a. results are presented in summary form in Table 4. This includes static and dynamic measurements.

**TABLE 4:** Institution A MPPG 5.a. result summary

Test	Comparison	Tolerance	Results
5.1	Dose distributions in planning module versus modeling (physics) module	Identical^a^	Pass
5.2	Dose in test plan versus clinical calibration condition^b^	0.50%	Pass
5.3	Dose distribution calculated in planning system versus commissioning data	2%	Pass
5.4	Small MLC‐shaped field (non‐SRS)	Table^c^	Pass
5.5	Large MLC‐shaped field with extensive blocking	Table^c^	Pass
5.6	Off‐axis MLC‐shaped field, with maximum allowed leaf over travel	Table^c^	Pass
5.7	Asymmetric field at minimal anticipated SSD	Table^c^	Pass
5.8	Field at oblique incidence (at least 20°)	Table^c^	Pass
5.9	Large (> 15 cm) field for each nonphysical wedge angle	Table^c^	Pass
6.1	Reported electron (or mass) densities against known values	–	Pass
6.2	Heterogeneity correction distal to lung tissue	3%	Pass
7.1	Small field PDD	Table^c^	Pass
7.2	Small MLC‐defined field output	2%	Pass
7.3	TG‐119 IMRT tests	TG‐218	Pass
7.4	Clinical tests	TG‐218	Pass
7.5	External review	IROC	Pass

^a^ Within the expected statistical uncertainty.

^b^ TPS absolute dose at reference point.

^c^ MPPG 5.a. Table 5.

#### 3.1.2 Tomo “Cheese” phantom

**FIGURE 1:**
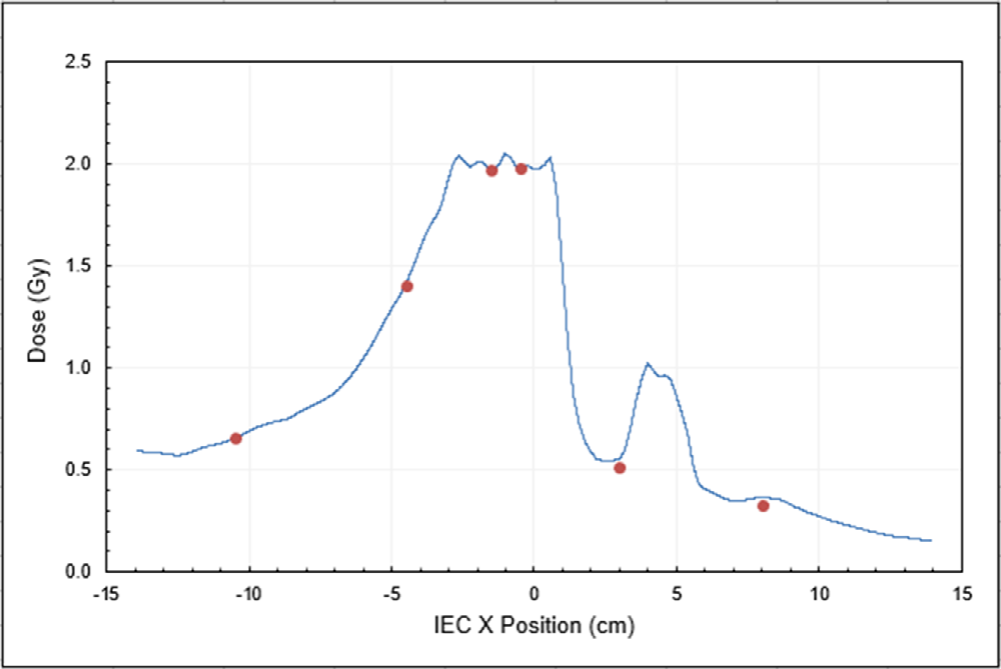
Calculated dose (line) and ion chamber (symbol) dose for a representative Tomo “Cheese” phantom measurement at Institution A for the TG‐119 C‐shape plan. The target in this case was within the –5 to 0 cm region. Error bars in both the horizontal and vertical are equal to the symbol diameter

A representative measurement using ion chambers in the Tomo “Cheese” phantom is shown in Figure 1, where calculated and measured doses are shown. Similar results were obtained for the remaining six VMAT plans created for four geometrically based and three anatomically based targets.

**FIGURE 2:**
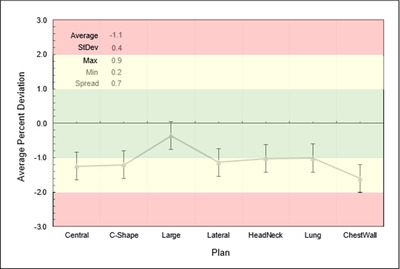
Output‐corrected ion chamber dose percent difference for all Tomo “Cheese” phantom measurements at Institution A. Each point is the average PD for all chambers in the high‐level, low‐gradient target region of the dose distribution for each plan, that is, those reading 90% of maximum dose or higher and within the target. Error bars are the average of all the standard deviations across all eligible ion chambers for all targets

Results using ion chambers in the Tomo “Cheese” phantom for the seven VMAT plans are shown in Figure 2, displaying calculated and measured dose percent differences. In this case, the calculated dose is the average dose to corresponding ion chamber structures lying within the target, and the graphed PD is the average across all ion chambers for a given plan.

#### 3.1.3 Delta4 phantom

**FIGURE 3:**
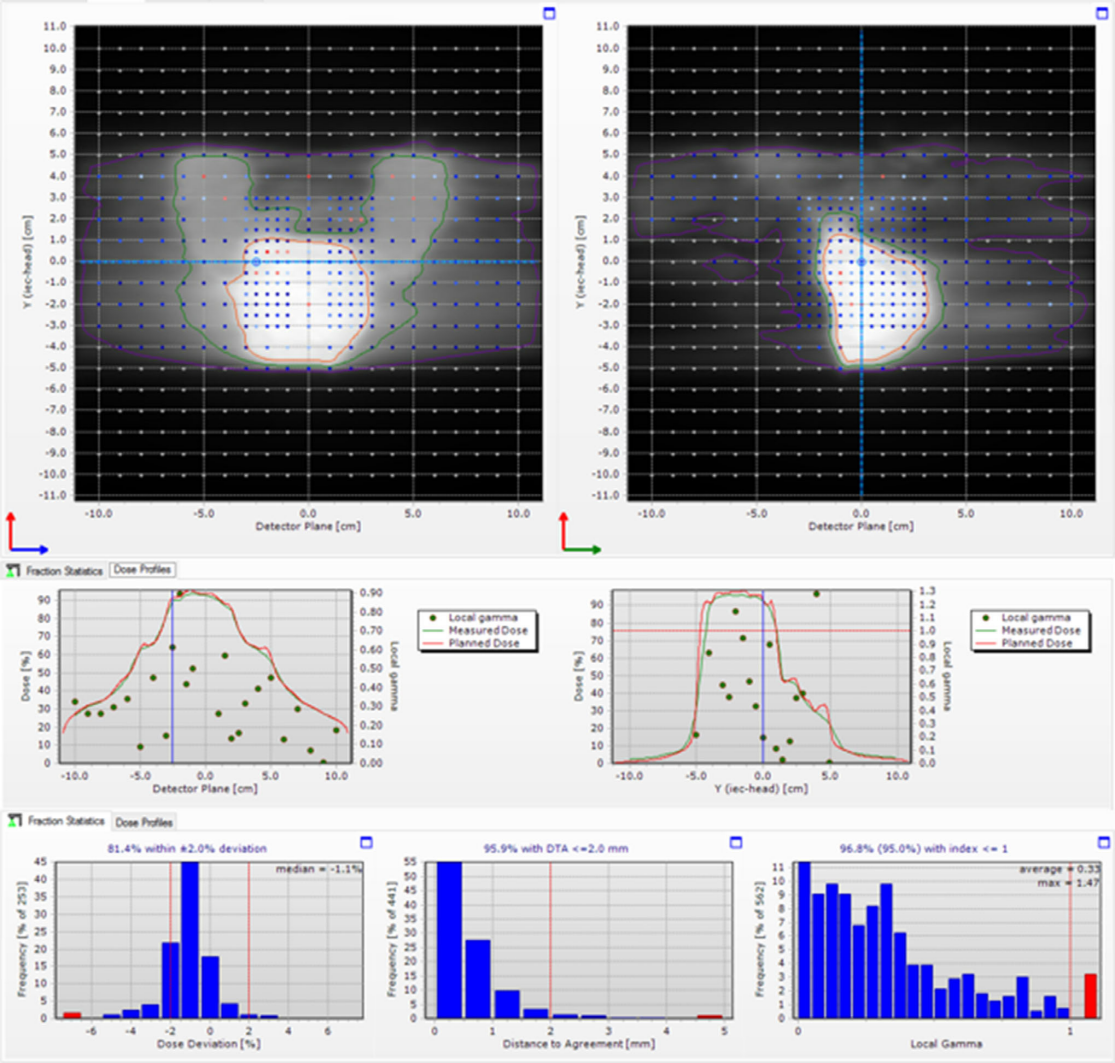
Representative Institution A Delta4 diode array result for a pelvis test plan (ID 09), including planar dose (top), line dose (middle), and median dose difference, distance‐to‐agreement, and gamma distributions (bottom, left to right). Gamma parameters are 2% local percent difference, 20% threshold, and 2 mm distance to agreement

Twenty‐five plans having target volumes of 15–2814 cm^3^ and equivalent sphere diameters of 3.1–17.5 cm were measured using the Delta4 device. Passing rates were 65–100% for the tighter gamma criteria (2% LPD, 2 mm DTA, and 20% DT), and 94–100% for clinical (3% GPD, 3 mm DTA, and 10% DT). The poorest performing targets were either a smaller highly modulated SBRT spine site or larger breast sites with target volumes lying near‐surface. These plans push the limits of the model and establish the boundary of model applicability. The average MDD was ‐1.1 ± 0.9%. A Delta4 result is shown in Figure 3 for a pelvis target (Plan ID 09), depicting typical level of agreement. Results from all plans are summarized in Table 5.

**TABLE 5:** Institution A Delta4 diode array anatomical test plan results

		Target	Sphere		Median dose		
	Site	volume	diameter	Delivery	Difference	Gamma (20% threshold)	
ID	name	(cm^3^)	(cm)	technique	(percent)	2%L/2 mm	3%G/3 mm
01	Lung	15.4	3.1	VMAT	−0.1	99.7	100.0
02	Brain	19.6	3.3	VMAT	−0.6	100.0	100.0
03	Brain	28.0	3.8	VMAT	−1.9	94.9	99.3
04	Spine	50.9	4.6	VMAT	−1.7	96.4	100.0
05	Prostate	51.7	4.6	VMAT	−1.3	96.4	100.0
06	Spine	54.4	4.7	VMAT	−3.8	84.8	93.7
07	Prostate	67.1	5.0	VMAT	−1.7	92.6	100.0
08	Headneck	120.9	6.1	VMAT	−1.5	92.8	99.8
09	Pelvis	178.3	7.0	VMAT	−1.0	96.3	100.0
10	Pelvis	188.6	7.1	VMAT	−1.3	97.2	100.0
11	Central cylinder	221.4	7.5	VMAT	−1.9	79.7	99.8
12	Off‐axis cylinder	221.5	7.5	VMAT	−1.4	95.4	99.3
13	C‐Shape	274.2	8.1	VMAT	−1.5	91.0	99.6
14	Brain	331.7	8.6	SAS	−0.8	99.8	100.0
15	Brain	360.3	8.8	VMAT	−0.3	99.8	100.0
16	Pancreas	380.1	9.0	VMAT	−1.0	96.2	99.9
17	Headneck	429.0	9.4	VMAT	−1.2	98.0	100.0
18	Lung	506.6	9.9	VMAT	−0.2	100.0	100.0
19	Breast	1288.1	13.5	VMAT	−1.6	65.0	95.5
20	Brain	1297.6	13.5	SAS	0.6	96.7	99.7
21	Breast	1504.7	14.2	VMAT	−0.9	92.5	100.0
22	Central cylinder	1621.7	14.6	VMAT	−1.4	93.5	100.0
23	Pelvis	2655.2	17.2	VMAT	−0.2	97.3	99.0
24	Pelvis	2815.1	17.5	VMAT	0.1	97.8	100.0
25	Breast	–	–	SAS	−1.6	65.0	95.5
–	No PTV structure			Average	−1.1	92.8	99.2
				StDev	0.9	9.6	1.7
				Min	−3.8	65.0	93.7
				Max	0.6	100.0	100.0
				Spread	4.4	35.0	6.3

Note: Plan ID 25 is a field‐in‐field tangent plan with no target volume. SAS is step‐and‐shoot IMRT delivery.

#### 3.1.4 Independent audit

The Institution A IROC Lung phantom results are summarized in Table 6.

**TABLE 6:** Independent audit results for Institution A

TLD location	IROC‐H versus institution	Criteria	Acceptable
PTV_TLD_sup	0.98	0.92–1.05	Yes
PTV_TLD_inf	0.99	0.92–1.05	Yes

Film plane	Gamma Index*	Criteria	Acceptable
Axial	99%	≥ 80%	Yes
Coronal	99%	≥ 80%	Yes
Sagittal	99%	≥ 80%	Yes
Average over three planes	99%	≥ 85%	Yes
*Percentage of points meeting gamma‐index criteria of 7% and 5 mm			

TLD location	Institution dose (cGy)	TLD dose (cGy)	Measured/institution
PTV_TLD_sup	2840	2788	0.98
PTV_TLD_inf	2823	2782	0.99

TLD location	Institution dose (cGy)	TLD dose (cGy)	Acceptability (cGy)*
HEART_TLD	213	222	27–417
CORD_TLD	257	261	66–456
*The 7% criterion is quoted as a percentage of the average PTV TLD doses and not as a local point comparison.			

### 3.2 Institution B

The results for Institution B are presented below. This includes MPPG 5.a. static beams measured with a 3D tank, and VMAT plans using an Octavius as well as ArcCHECK devices.

#### 3.2.1 MPPG 5.a. summary

All MPPG 5.a. results are presented in summary form in Table 7. This includes static and dynamic measurements.

**TABLE 7:** Institution B MPPG 5.a. measurement result summary

Test	Comparison	Tolerance	Results
5.1	Dose distributions in planning module versus modeling (physics) module	Identical^a^	Pass
5.2	Dose in test plan versus clinical calibration condition^b^	0.50%	Pass
5.3	Dose distribution calculated in planning system versus commissioning data	2%	Pass
5.4	Small MLC‐shaped field (non‐SRS)	Table^c^	Pass
5.5	Large MLC‐shaped field with extensive blocking	Table^c^	Pass
5.6	Off‐axis MLC‐shaped field, with maximum allowed leaf over travel	Table^c^	Pass
5.7	Asymmetric field at minimal anticipated SSD	Table^c^	Pass
5.8	Field at oblique incidence (at least 20°)	Table^c^	Pass
5.9	Large (> 15 cm) field for each nonphysical wedge angle	Table^c^	Pass
6.1	Reported electron (or mass) densities against known values	–	Pass
6.2	Heterogeneity correction distal to lung tissue	3%	Pass
7.1	Small field PDD	Table^c^	Pass
7.2	Small MLC‐defined field output	2%	Pass
7.3	TG‐119 IMRT tests	TG‐218	Pass
7.4	Clinical tests	TG‐218	Pass
7.5	External review	IROC	Pass

^a^ Within the expected statistical uncertainty.

^b^ TPS absolute dose at reference point.

^c^ MPPG 5.a. Table 5.

#### 3.2.2 Octavius phantom

**FIGURE 4:**
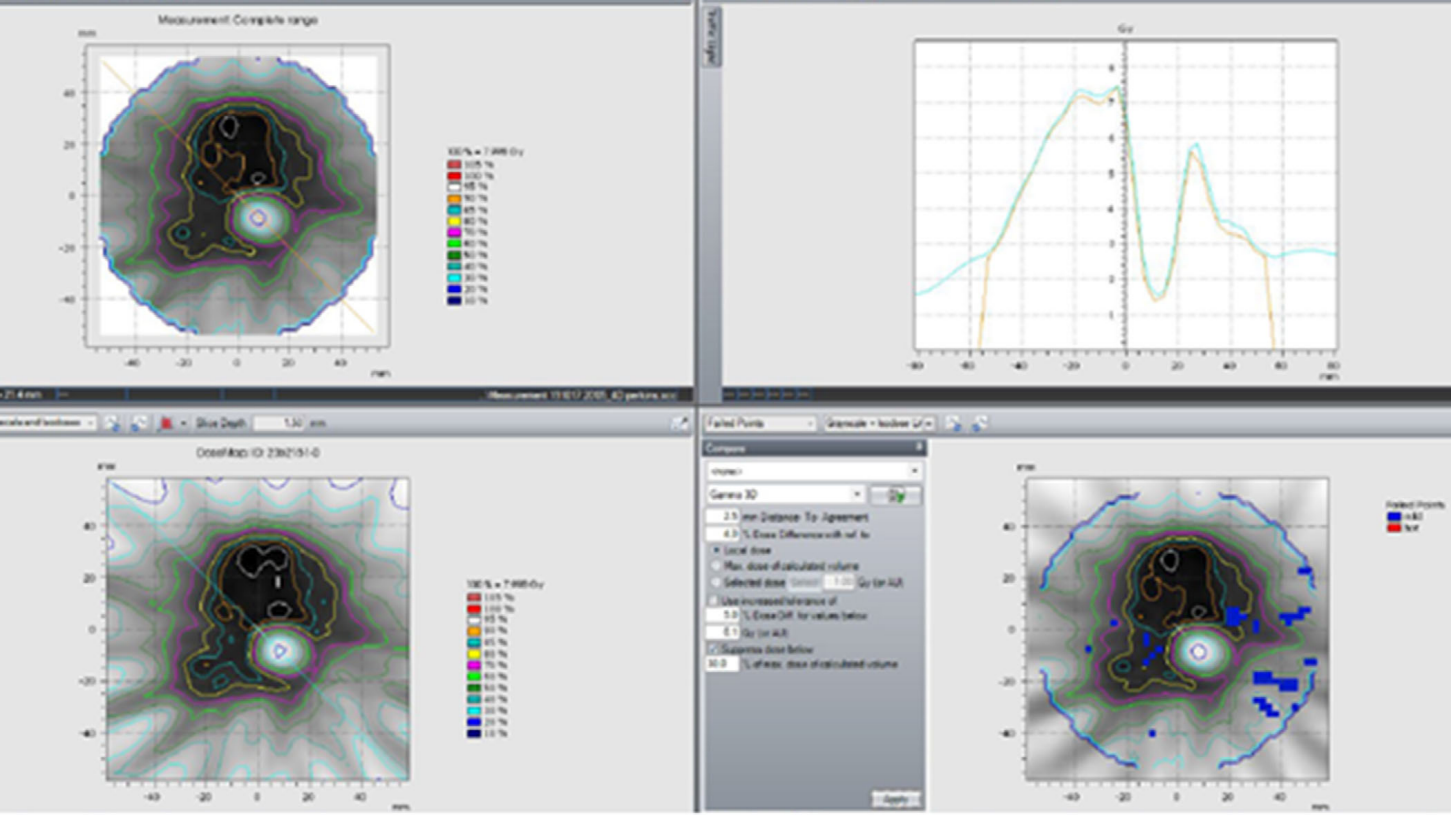
Representative Octavius diode array result from Institution B featuring isodose line displays for the measured dose distribution (top‐left), planned dose distribution (bottom‐left), a profile comparison (top‐right), and gamma analysis (bottom‐right)

Twenty SSRS spine plans were measured using the Octavius phantom. A representative analysis is shown in Figure 4, and the results are summarized in Table 8. Passing rates were 96.8 ± 2.8% for the tighter criteria (2% LPD, 2 mm DTA, and 30% DT) and were 99.6 ± 0.5% for the clinical ones (4% LPD, 2.5 mm DTA, and 30% DT). The MDD as a percentage of the maximum plan dose is –1.7 ± 0.5%.

**TABLE 8:** Institution B Octavius results

		Target	Sphere		Median dose		
	Site	volume	diameter	Delivery	Difference	Gamma (10% threshold)	
ID	name	(cm^3^)	(cm)	technique	(percent)	2%L/2 mm	4%L/2.5 mm
01	Brain	14.2	3.0	VMAT	−1.9	99.6	100.0
02	Brain	18.5	3.3	VMAT	−1.9	99.8	100.0
03	Brain	23.8	3.6	VMAT	−1.3	99.2	99.9
04	Brain	25.5	3.7	VMAT	−1.4	98.8	100.0
05	Brain	27.8	3.8	VMAT	−0.7	97.6	99.8
06	T‐Spine	29.9	3.9	SAS	−2.5	97.8	99.7
07	T‐Spine	35.0	4.1	SAS	−2.4	94.2	99.0
08	Brain	37.3	4.1	VMAT	−1.7	100.0	100.0
09	Brain	42.2	4.3	VMAT	−2.5	99.3	100.0
10	Brain	44.7	4.4	VMAT	−2.1	96.2	99.9
11	C‐Spine	52.5	4.6	SAS	−1.2	94.4	99.3
12	Brain	53.9	4.7	VMAT	−1.4	96.5	100.0
13	Brain	54.3	4.7	VMAT	−1.1	99.6	100.0
14	T‐Spine	63.3	4.9	SAS	−1.0	97.0	99.4
15	L‐Spine	81.7	5.4	SAS	−1.3	95.5	99.6
16	T‐Spine	93.5	5.6	SAS	−2.1	97.3	99.7
17	T‐Spine	141.2	6.5	SAS	−1.5	96.4	99.5
18	C‐Spine	146.3	6.5	SAS	−1.6	92.4	98.7
19	T‐Spine	199.8	7.3	SAS	−1.8	96.3	99.8
20	L‐Spine	406.2	9.2	SAS	−2.2	89.0	98.4
				Average	−1.7	96.8	99.6
				StDev	0.5	2.8	0.5
				Min	−2.5	89.0	98.4
				Max	−0.7	100.0	100.0
				Spread	1.9	11.0	1.6

Note: SAS is step‐and‐shoot IMRT delivery.

#### 3.2.3 ArcCHECK phantom

**FIGURE 5:**
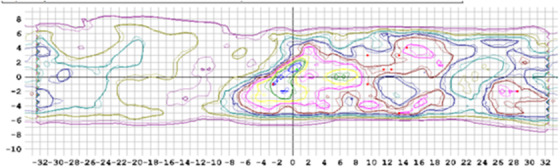
Representative ArcCHECK diode array result from Institution B showing an isodose comparison between the measured and planned dose distributions. Red dots indicate points failing Gamma with a measured dose higher than calculated and the blue dots indicate points failing with lower than calculated dose

Twenty SBRT lung and abdominal plans and 20 nonstereotactic plans were measured using the ArcCHECK. A representative analysis is shown in Figure 5, and the results are summarized in Table 9. Passing rates were 99.2 ± 1.1% for clinical criteria (3% GPD, 3 mm DTA, and 10% DT). It was not possible to reanalyze the data with stricter criteria, and the ArcCHECK software does not report MDD information.

**TABLE 9:** Institution B ArcCHECK results

		Target	Sphere		Median dose		
	Site	volume	diameter	Delivery	Difference	Gamma (10% threshold)	
ID	name	(cm^3^)	(cm)	technique	(percent)	2%L/2 mm	3%G/3 mm
01	Thoracic SBRT	2.5	1.7	VMAT	–	–	99.1
02	Head & Neck SBRT	3.0	1.8	VMAT	–	–	97.7
03	Thoracic SBRT	9.3	2.6	VMAT	–	–	99.6
04	Thoracic SBRT	10.4	2.7	SAS	–	–	99.7
05	Thoracic SBRT	11.0	2.8	VMAT	–	–	99.2
06	Thoracic SBRT	12.3	2.9	VMAT	–	–	99.2
07	Thoracic SBRT	12.5	2.9	VMAT	–	–	99.0
08	Thoracic SBRT	15.5	3.1	VMAT	–	–	99.6
09	GI SBRT	17.3	3.2	SAS	–	–	96.5
10	Eye	19.5	3.3	VMAT	–	–	100.0
11	Thoracic SBRT	20.6	3.4	SAS	–	–	100.0
12	Thoracic SBRT	23.8	3.6	SAS	–	–	99.7
13	Thoracic SBRT	25.9	3.7	VMAT	–	–	99.7
14	Thoracic SBRT	35.1	4.1	VMAT	–	–	99.2
15	Head & Neck	36.2	4.1	VMAT	–	–	100.0
16	Head & Neck	37.8	4.2	VMAT	–	–	96.1
17	Thoracic SBRT	39.4	4.2	VMAT	–	–	100.0
18	Thoracic SBRT	40.0	4.2	VMAT	–	–	98.0
19	GI SBRT	50.3	4.6	SAS	–	–	100.0
20	Thoracic SBRT	56.4	4.8	VMAT	–	–	99.7
21	Brain	69.7	5.1	VMAT	–	–	95.5
22	GI SBRT	73.5	5.2	SAS	–	–	99.4
23	Brain	97.9	5.7	VMAT	–	–	99.4
24	Brain	109.3	5.9	VMAT	–	–	99.3
25	GI SBRT	112.3	6.0	SAS	–	–	98.9
26	Head & Neck	126.3	6.2	VMAT	–	–	96.9
27	Brain	163.3	6.8	VMAT	–	–	99.3
28	Brain	164.5	6.8	VMAT	–	–	99.8
29	Lung	184.5	7.1	VMAT	–	–	100.0
30	Brain	193.1	7.2	VMAT	–	–	100.0
31	Brain	197.3	7.2	VMAT	–	–	100.0
32	Brain	200.4	7.3	VMAT	–	–	99.8
33	Brain	254.5	7.9	VMAT	–	–	99.3
34	Brain	304.7	8.3	VMAT	–	–	99.3
35	Brain	347.9	8.7	VMAT	–	–	100.0
36	Brain	350.9	8.8	VMAT	–	–	100.0
37	GI SBRT	380.6	9.0	SAS	–	–	98.9
38	Lung	456.6	9.6	VMAT	–	–	99.9
39	Brain	486.8	9.8	VMAT	–	–	99.9
40	GI	528.7	10.0	VMAT	–	–	100.0
				Average	–	–	99.3
				StDev	–	–	1.1
				Min	–	–	95.5
				Max	–	–	100.0
				Spread	–	–	4.5

Note: SAS is step‐and‐shoot IMRT delivery.

#### 3.2.4 Independent audit

The Institution B IROC Lung phantom results are summarized in Table 10.

**TABLE 10:** Independent audit results for Institution B

TLD location	IROC‐H versus institution	Criteria	Acceptable
PTV_TLD_sup	0.99	0.92–1.05	Yes
PTV_TLD_inf	0.98	0.92–1.05	Yes

Film plane	Gamma Index*	Criteria	Acceptable
Axial	98%	≥ 80%	Yes
Coronal	97%	≥ 80%	Yes
Sagittal	96%	≥ 80%	Yes
Average over three planes	97%	≥ 85%	Yes
*Percentage of points meeting gamma‐index criteria of 7% and 5 mm			

TLD location	Institution dose (cGy)	TLD dose (cGy)	Measured/institution
PTV_TLD_sup	630	622	0.99
PTV_TLD_inf	629	615	0.98

TLD location	Institution dose (cGy)	TLD dose (cGy)	Acceptability (cGy)*
HEART_TLD	68	64	21–107
CORD_TLD	82	72	35–122
*The 7% criterion is quoted as a percentage of the average PTV TLD doses and not as a local point comparison.			

## 4 DISCUSSION

In RayStation, the calibration coefficient and output factors scale the input measured dose curve data, while the OFCs and normalization coefficient scale the dose calculation. Comparing the ratios in Tables 2 and 3, as well as those stated in the Results section, we see everything agrees to within 0.5% or better (except the 40 × 40 cm^2^ field size). One could argue that Institution B did not need to update the Institution A values with their own. Comparing the IMRT/VMAT QA results between institutions, there appears to be a systematic scaling offset for dynamic (VMAT) plans, as the MDD was in the neighborhood of –1% across the board. A future revision of the current model should take this scaling into account. We point out, however, that this consistency in the amount of offset actually points to the robustness of the model's performance across a broad plan and measurement spectrum.

Commissioning a TPS in an MVE is one of the most challenging and time‐intensive tasks a clinical medical physicist can perform. In an SVE, the physicist has an option to accept a vendor‐provided model with some confidence. In that case, the task mainly is acceptance with few if any adjustments. In an MVE, the physicist is often faced with the challenge of having neither a matched machine nor an optimal preconfigured clinical model. The goal of this work is to demonstrate that a physicist in a multi‐vendor environment can have the same experience as they would in a single‐vendor environment.

Another challenge in commissioning is in the TPS itself. Developing and validating a clinical model requires an understanding of the TPS representation of the real‐world machine and how the model parameters affect dose calculation in clinical situations. Parameter values ideally should begin with real‐world (physical) inputs, but often these values do not result in an acceptable clinical model in part due to simplifications made in the TPS representation of the physical machine. Two examples in RayStation are representing beam‐limiting devices, such as MLCs and jaws as having zero height, or the use of nontilting dose kernels. The model parameter values must be tuned to accommodate these algorithmic assumptions and implementation approximations in the actual TPS dose calculation engine.

Clinical model development is rife with pitfalls. A clinical model contains a large number of parameter values that are needed to ensure dose calculation accuracy over a wide range of delivery scenarios. This poses a significant challenge to identify a set of values which is accurate and robust to a wide spectrum of treatment plans. This is because parameter values are coupled, that is, the optimal value of any one is dependent on one or more others. As these parameter values move away from physically based ones, it becomes more likely that the clinical model will land in one of many mostly indistinguishable local minima. In this situation, “reasonable” parameter value adjustments do not improve the model accuracy, and the likelihood of finding the parameter values that will result in a better clinical model is limited.

Clinical model accuracy is influenced by a number of factors. These include limitations in the measured beam data, quality and implementation of patient‐specific QA devices, as well as tools within the TPS, all which can be significantly exacerbated by capabilities and experience of people involved with these. Measured scan beam data are limited in quality due to the trade‐off in noise, detector resolution, and mechanical positioning. Model optimization using a routine QA device requires additional independent devices for validation, which may not be at every institution's disposal. Lastly, missing tools in the TPS for model parameter value optimization, especially for MLCs, hinder optimization of those parameter values. Koger et al. pointed out the associated pitfalls when the software does not provide such tools.^23^ When significant work needs to be performed outside of the TPS, this can be a burdensome effort that entails significant tool development by the end user.

As IROC data clearly show, there is wide variation in clinical model performance across their surveyed institutions, suggesting this exists within the broader radiation therapy community.^7^ Their surveys indicate that for any given TPS, there is a wide spectrum of model parameter values being used clinically.^1^ All of these shortcomings in a locally developed model can be addressed with the utilization of a portable preconfigured and optimized model.

Many vendors have been able to standardize TDS performance to the degree that TDSs of a similar model can meet the same very tight performance specifications, and TPS representations employed by most vendors are able to create accurate and reliable clinical models. This leads one to the conclusion that the main variability is from the people driving the technology. The people creating the model affect the tuning of model parameter values, affect measuring the input beam data, and influence defining what is acceptable. It is much more probable now that variation in measured beam data is due to variation in equipment setup and data acquisition, not variation in TDS performance. Consequently, the quality of the clinical model is driven by the quality of the beam data used to optimize the model. If one is having difficulties developing an acceptable clinical model, it behooves the user to verify the quality of the measured beam data. In the past, machine performance, as well as measurement equipment variability obscured the role that individuals played in the existence of differing beam models. Now, the variation's greatest influence is not due to technology, but its use.

A critical point is that any model parameter value tuning needs to be performed against measurement using well‐established, absolutely calibrated devices. Care must be taken not to build measurement device uncertainty into a clinical model. Therefore, final validation should be performed against different, well‐established, absolutely calibrated devices. More confidence is gained when a wider variety or broader spectrum of devices is utilized. In this work, the measurement devices span multiple vendors and two institutions.

Model validation is a significant and time‐consuming exercise distinct from model parameter value optimization, the latter which can consume most of the allotted time, leading to reduced time available for validation. Using a template model relieves this pressure significantly, allowing for more extensive plan‐based validation across a broader spectrum of test plans. In addition, at many institutions, there is at most one QA device available, preventing independent validation of an in‐house developed model, which is critical. Although an independent audit, for example, using a service such as IROC, can help satisfy independent validation requirements, their tests often serve as a basic check designed to catch gross errors, and cannot be a replacement for a second independent device. A template that has undergone validation on multiple machines and QA devices can significantly reduce the above risks to or with necessary local model validation.

A clinical model must pass validation on a number of levels. First, it must be able to reproduce the input beam data and meet institutional standards. Next, the beam model must accurately calculate dose for simple field geometries. Then, the model must perform well in clinically relevant scenarios. This should involve the use of a suite of test plans specific to the spectrum of an institution's planning approach and treatment site methodology. The model should pass the common clinical gamma test with typical metric (e.g., 3% GPD, 2 mm DTA, and 10% DT criteria), and also be tested using more stringent criteria (e.g., 2% LPD, 2 mm DTA, and 20% DT) to reveal where the model begins to break down. Finally, the model should pass an audit, preferably with an independent organization, such as IROC.

Our results in this work focused on the validation of a single portable TrueBeamSTx model. All of the above considerations earlier in this section apply. First, Institution A performed requisite parameter value optimization and independent validations, that is, the extensive work any institution in a mixed‐vendor environment would do if they were starting from scratch. Validations were performed with two completely different measurement systems by a number of staff over an extended period. An independent IROC audit was also passed. This then set the stage for Institution B to consider utilizing the Institution A Model as‐is and proceeding directly to MPPG 5.a. validation. Special attention was paid to measurement‐based IMRT/VMAT QA for clinical cases representative of those treated at Institution B. We note that Institution B had independently and in parallel developed their own clinical model with similar significant effort. However, the portable one from Institution A performed slightly better, and they opted to go live with the latter. Adoption of the Institution A (source) model at Institution B required no additional time optimizing the latter model and allowed more time for validating.

The current work demonstrates that it is possible to use a single class‐level mixed‐vendor solution at two completely different institutions. The same RayStation beam model for a TrueBeamSTx flattened beam, with minimal changes in output parameter values, was successfully validated for patient care using similar guidance, namely, MPPG 5.a., but for different TPS and TDS instances, as well as differing equipment, measurement methodologies, and personnel. The validation results point to the feasibility of a mixed‐vendor TPS model interinstitutional portability. There is a significant operational impact, as with a faster TPS/TDS implementation, a faster turn‐around can save significant resources while at the same time ensuring high quality.

## 5 CONCLUSION

We have validated that a single beam model can be used for three c‐arm TDSs (Linacs) that are of the same model and at two completely independent institutions. They have not been explicitly matched to each other, but meet the same vendor performance specifications. This indicates that, without any parameter value optimization work, it is possible to meet or exceed all MPPG5.a guidelines and TG‐218 criteria. This was achieved across a number of different measurement devices and planning techniques, thereby indicating robustness of the model and broad applicability. Developing a suite of portable beam models will improve the process of TPS implementation. This opens up the possibility of more accurate and uniform dose modeling across the community.

## FUNDING

This research did not receive any specific grant from funding agencies in the public, commercial, or not‐for‐profit sectors.

## CONFLICTS OF INTEREST

The authors have no conflicts of interest to report.

## AUTHOR CONTRIBUTIONS

All listed authors above contributed equally to the intellectual content of the manuscript, including the design, acquisition, analysis, and interpretation. They participated equally in drafting, revising, and interpreting the material. All authors have read and approved the final submitted version of the manuscript. Additionally, none of the authors have any outside funding sources, which contributed to this work, and have not published the data or results prior.
